# The expansion of newborn neurons in hippocampus improves social recognition deficit in a mouse model of autism

**DOI:** 10.3389/fpsyt.2023.1162179

**Published:** 2023-05-05

**Authors:** Hu Meng, Qiongwei Li, Jinxin Wang, Weihua Yue, Dai Zhang, Xiaoxuan Sun, Lifang Wang, Jun Li

**Affiliations:** ^1^Peking University Sixth Hospital, Peking University Institute of Mental Health, NHC Key Laboratory of Mental Health (Peking University), National Clinical Research Center for Mental Disorders (Peking University Sixth Hospital), Beijing, China; ^2^State Key Laboratory of Cognitive Neuroscience and Learning, IDG/McGovern Institute for Brain Research, Beijing Normal University, Beijing, China; ^3^PKU-IDG/McGovern Institute for Brain Research, Peking University, Beijing, China; ^4^Institute for Brain Research and Rehabilitation (IBRR), Guangdong Key Laboratory of Mental Health and Cognitive Science, South China Normal University, Guangzhou, China

**Keywords:** autism, social recognition, dentate gyrus, neurogenesis, treatment

## Abstract

**Introduction:**

Autism spectrum disorders (ASDs) are a group of neurodevelopmental disorders characterized by core symptoms of impaired social interaction and communication. The pathological mechanism and treatment are not clear and need further study. Our previous study found that the deletion of high-risk gene Autism Susceptibility 2 (AUTS2) in mice led to dentate gyrus (DG) hypoplasia that highly associated with impaired social novelty recognition. Here we aim to improve the social deficit through increasing the neurogenesis in the subgranular zone (SGZ) and expanding the newborn granule neurons in DG.

**Methods:**

Three approaches including repeated oxytocin administration, feeding in enriched environment and overexpression of cyclin-dependent kinase 4 (Cdk4)-CyclinD1 complex in DG neural stem cells (NSCs) at the post-weaning stage were conducted.

**Results:**

We found that the number of EdU labeled proliferative NSCs or retrovirus labeled newborn neurons was significantly increased after manipulations. The social recognition deficit was also significantly improved.

**Discussion:**

Our findings suggested a possible strategy to restore the social deficit through expansion of newborn neurons in hippocampus, which might provide a new insight into the treatment of autism.

## Introduction

Autism spectrum disorders (ASDs) are a group of neurodevelopmental disorders mainly characterized by social interaction and communication deficits, and repetitive and stereotyped behaviors, of which social deficit is the core symptom (1). At present, the pathogenesis of ASDs is not clear. Twin study reported the heritability of ASDs as about 88–97%, indicating that the genetic factors play an important role in the etiology of the disease (2). The identified high-risk genes cause abnormal neurodevelopment of ASDs (3, 4), resulting in abnormal behaviors including social deficit (5, 6). For example, numerous studies on neuroimaging (7), neuropathology (8, 9) and neurobiology (10) have found that the structure and/or function of dentate gyrus (DG) were affected in patients with ASDs or autistic animal models with susceptibility gene variations, indicating the possibility that DG hypoplasia was involved in the etiology of ASDs. However, whether DG hypoplasia is a potential treatment target for ASDs need to be further investigated.

Autism susceptibility2 (AUTS2) is a high-risk gene for ASDs. Our previous study found that the *Auts2^fl/fl;Emx1-Cre^* mice showed DG hypoplasia that highly associated with social novelty recognition deficit (11). Although the activation of supramammillary nucleus (SuM)-DG-CA3 neural circuit could improve the impaired social novelty preference in mice, the employed optogenetic and chemogenetic approaches still have more work to be done before they can be applied in clinical treatment. Other forms of non-invasive neuromodulation, such as transcranial magnetic stimulation, have difficulties in targeting the deeper, subcortical structures or specific cell types (12). These techniques also still need more research foundation and technology development. In addition, most of the approved medications show benefit only on irritability, aggression, repetitive behaviors and insomnia, but not social skills in patients with ASDs (6). Therefore, the novel strategy for treatment of social deficit needs to be explored.

Newborn neurons have been found in the brains of rodents (13), primates (14), and humans (15) at the postdevelopmental stage. The subgranular zone (SGZ) of DG and the subventricular zone (SVZ) of lateral ventricles are the major studied and considered neurogenic zone in nearly all mammals (16), while the olfactory bulb (OB) also contain neurogenesis sites in the rodent brain (17). Previous studies have shown that newborn neurons in the hippocampus play a critical role in cognitive function, emotion and social interaction (18, 19). Social isolation or exposure to harmful environment at early postnatal stage leads to the impairment of hippocampal neurogenesis and social deficits (20, 21). However, it is not clear whether expansion of newborn neurons through enhancing the hippocampal neurogenesis could be considered as a novel intervention target to improve the social deficit in autistic animal model with DG hypoplasia.

Repeated oxytocin administration, feeding in environment enrichment and genetic overexpression of cyclin-dependent kinase 4 (Cdk4) and CyclinD1 had been proved to trigger the hippocampal neurogenesis in adult mouse brain (22–24). The aim of present study is to improve the social deficit through increasing the proliferation of the neural stem cells (NSCs) in SGZ of DG and expansion of newborn neurons through these approaches at the post-weaning stage. Our findings might provide a new insight in the treatment of ASDs through the strategy of neurogenesis regulation.

## Materials and methods

### Mouse

As previously described (11), *Auts2^fl/fl^* mice were generated by Biocytogen (Beijing, China). The Emx1-Cre mice were gifts from Prof. C. Zhao (Southeast University, Nanjing, China). All mouse strains used in the present study are on C57BL/6 background. All animal procedures were approved by the Animal Ethical Committee of Peking University Health Center. The transgenic mice were genotyped by PCR as follows. For *Auts2^fl/fl^* mice, the oligos included forward primer 5′-TCCTCGTTTGAATTGACCG-GTG-3′ and reverse primer 5′-TGGTTTATGGCTCTAACGCCGTG-3′; for Cre recombinase, forward primer 5′-CCGCAGAACCTGAAGATG-3′ and reverse primer 5′-GCTACACCAGAGAC-GGAA-3′. the PCR products were 194 base pairs (bp; wild-type allele) and 323 bp (LoxP allele) for *Auts2*; and 499 bp for Cre recombinase. The PCR program included 95°C for 5 min (1 cycle); 95°C for 30 s, 59°C for 30 s, and 72°C for 45 s (35 cycles); and 72°C for 10 min (1 cycle). The mice used for EdU labeling and behavioral test were male.

### EdU labeling

After the three manipulations, a part of the mice were used for cell proliferation analysis. Mice were intraperitoneally injected with a 50 μg/g body weight dose of 5-ethynyl-2′-deoxyuridine (EdU). After 4 h, mice were perfused and fixed in fresh 4% PFA at 4°C for 24 h. Frozen sections for the visualization of EdU-labeled cells were reacted by the Click-iT Plus EdU Kit (Invitrogen, C10640, United States) with Alexa Fluor 647 azide for 30 min.

### Drug administration

Oxytocin powder (Guoping Pharmaceutical, Hefei, Anhui Province, China) dissolved in saline to 0.1 mg/mL. At the age of 3 weeks, oxytocin was administered by intraperitoneal injection with 1 mg/kg daily for 2 weeks. Doxycycline (Sigma-Aldrich), a structural isomer of tetracycline, was dissolved in drinking water with the final concentration of 75 mg/mL. Two weeks after virus injection, doxycycline was administration through drinking water for another 2 weeks.

### Environment enrichment

Briefly, 10 mice were housed in environment enriched large customized cages (400 mm length × 250 mm width × 200 mm height), which consisted a big plastic shelter, a cardboard roll, a plastic tube, a ladder, three toys, a running wheel, and a spice box with two different scents of spices are replaced in turn every day. Over the cage, a stainless steel grid with foods and a bottle of water. According to the experimental procedure, three-week-old mice were maintained under enriched conditions for 3 weeks.

### Tet-off system and Virus injection

According to Gossen and Bujard (25), the Tet-off system are based on two crucial elements, tetracycline-controlled transactivator (tTA) and tTA-Dependent Promoter. To generate promoters activatable by tTA, tetracycline operator (tetO) were inserted upstream of promoter sequences. In the absence of tetracycline, the tTA will bind to tetracycline (Tet) response (tetR) element and activate the region to promote expression. In the presence of tetracycline, the combination of tetracycline and tTA prevents the combination of tTA and tetR sequence, resulting in the silent of downstream gene. Retrovirus (26) were constructed in OBIO Technology (Shanghai, China) based on the above theory. pROV1: pROV-EF1α-tTA-P2A-EGFP-WPRE; pROV2: pROV-TRE-EF1α-Cdk4-HA-P2A-CyclinD1-3FLAG-WPRE. The mouse was anesthetized with pentobarbital sodium (70 mg/kg of body weight), and the virus was delivered using a 5-μL syringe with a thin 34-gauge metal needle (Hamilton Instruments) at the rate of 80 nL/min. The retrovirus were microinjected into DG region (1 μL per site, 0.5 μL for each virus; diluted from 0.5–1 μL with saline for single virus injection) on the left and right hippocampus [anterior–posterior (AP), −2.3 mm; medio-lateral (ML), ± 1.5 mm; dorsoventral (DV), −2.5 mm from the bregma], the representative images were showed in Supplementary Figure 2.

### Cell culture

For cell culture, the Human embryonic kidney (HEK) 293 cells were maintained in High Glucose Dulbecco’s Modified Eagle Medium (DMEM, Hyclone, SH30022.01, United States) containing 10% fetal bovine serum (FBS, GIBCO, 10099-141C, United States). For virus transfection, cells were inoculated into 6-wells plates which contained 1 mL culture medium. The retrovirus was added with the volume of 5 μL for each Retrovirus (5 × 10^8^ virus titer). After 12 h, the medium was supplemented to 2 mL with fresh DMEM containing 10% FBS. Then Doxycycline was added in the culture medium with the final concentration of 1 or 10 μg/mL, and cultured for another 48 h for harvest.

### Western blot

Tissues or cells were homogenized with lysis buffer [50 mM tris (pH 7.4), 150 mM NaCl, 1% Triton X-100, and 0.1% SDS] with protease inhibitors (Roche, 11,697,498,001). Lysates were centrifuged at 16,000 g for 30 min. The supernatants were collected and boiled with 4× Laemmli sample buffer (Sigma-Aldrich) and loaded onto 10% SDS–polyacrylamide gel electrophoresis gels (Invitrogen). After transmembrane, the membranes were blocked at room temperature for 60 min in 5% nonfat drymilk in phosphate-buffered saline Tween (PBST) and then incubated with primary antibodies overnight: rabbit anti-HA (1:1,000; Cell Signaling Technology, 3724S), rabbit anti-FLAG (1:1,000; Cell Signaling Technology, 2368S), rabbit anti-GFP (1:1,000; Cell Signaling Technology, 2956S), and rabbit anti-Tubulin (1:1,000; Abcam, ab108629). After washing with PBST for three times, the horseradish peroxidase–conjugated secondary antibodies (1:2,000; Cell Signaling Technology) were incubated at room temperature for 1 h. Membranes were imaged using an enhanced chemiluminescence reagent (Thermo Fisher Scientific) and a Tanon 5200 Automatic chemiluminescence imaging analysis system (Tanon, Shanghai, China).

### Three-chamber social interaction test

After the three manipulations, mice were used for social interaction test. The social testing arena was a three-chambered box. Each chamber was 20 cm by 40 cm by 22 cm in size with a rectangular openings (5 cm by 8 cm), allowing access into each chamber. The enclosures have an internal diameter of 7 cm and a height of 15 cm; the grid bars are 5 mm in diameter and are 10 mm apart, allowing nose contact through the bars but preventing fighting. Target mice were 6-week old gender matched mice that had been habituated by being placed inside the grid enclosure for 3 days before the beginning of testing. Test mice were habituated to the testing room for at least 30 min before the start of behavioral tasks. In the first session, the test mouse was placed in the middle chamber, allowing free exploration in three chambers for a 5-min habituation. In the second session (Trial 1), unfamiliar mouse Stranger 1 (S1) that had had no prior contact with the test mouse was placed in one of the enclosures. The sliding doors were opened, and the test mouse was allowed to explore the entire social test arena for a 10-min session. In the third session (Trial 2), another unfamiliar mouse Stranger 2 (S2) was placed into the previously empty enclosure. The test mouse had a choice between the first, already-investigated mouse S1 and the novel, unfamiliar mouse S2. The mouse was recognized by the EthoVision 7.0 video tracking system (Noldus, Netherlands). The amount of time spent in each chamber and the time spent in close interaction (with the nose point within 2 cm of the enclosure) were recorded and analyzed.

### Statistical analysis

During testing and counting, the experimenters were blinded to the genotype and treatment of the mice. Samples size (n) was indicated in the figure legends. No samples or animals were excluded unless specified in the experimental procedure. All data in accordance with the normal distribution were represented as mean ± SEM. The homogeneity test of variance was performed before the statistical test. Statistical Package for the Social Sciences 25.0 (SPSS, Chicago, IL, United States) and GraphPad Prism 7.04 were used for statistical analysis. *p* < 0.05 was considered as statistical significance. More statistical details are displayed in the Supplementary Table 1.

## Results

### Post-weaning treatment of oxytocin promotes the proliferation of NSCs in DG and reverses social deficit in *Auts2^fl/fl;Emx1-Cre^* mice

**Figure 1:**
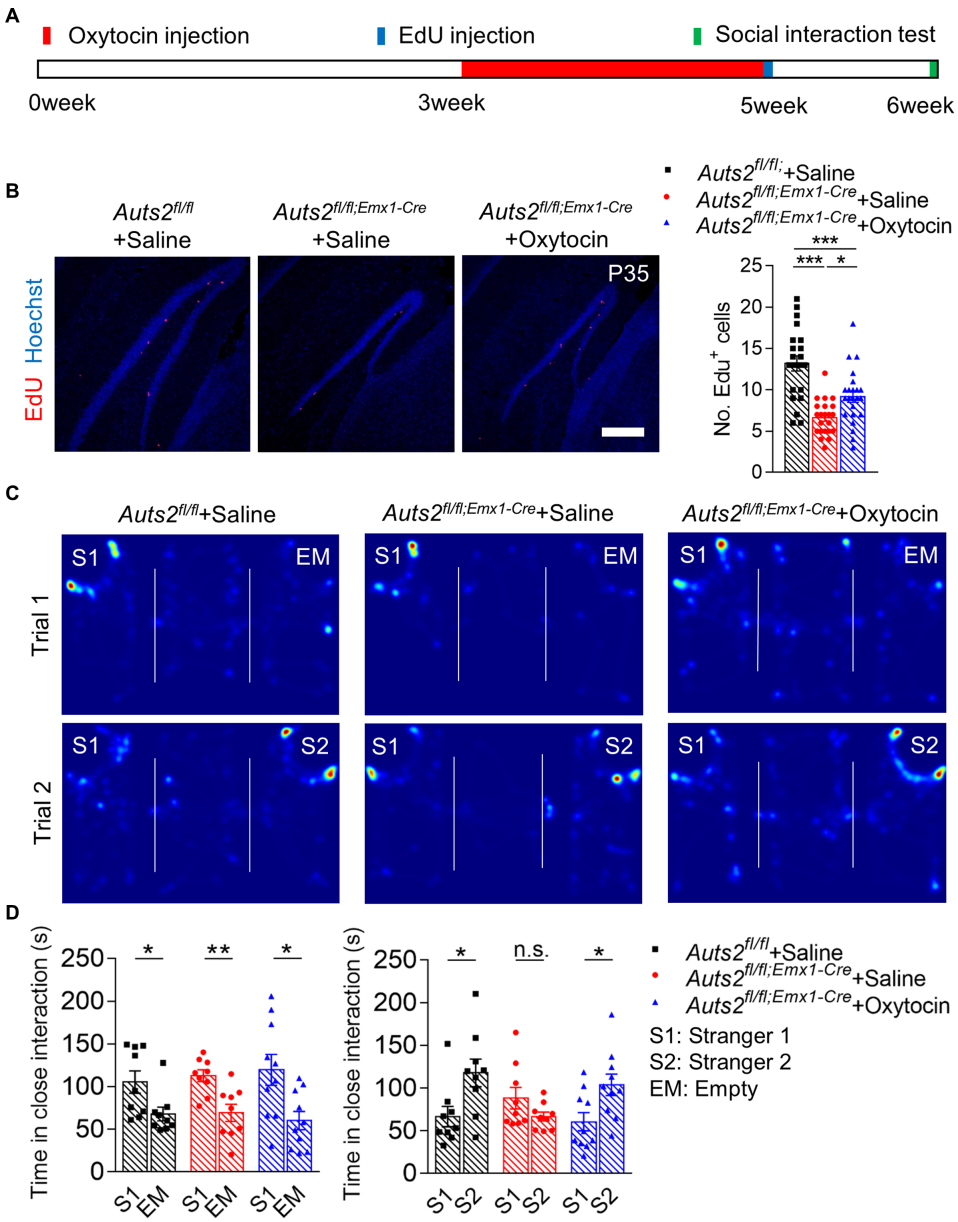
Post-weaning treatment of Oxytocin promotes the proliferation of NSCs in DG and reverses social deficit in *Auts2^fl/fl;Emx1-Cre^* mice **(A)** Experimental strategy of oxytocin treatment (1 mg/kg/mice, once per day from 3 week to 5 week), proliferation analysis and social behavioral test. **(B)** Cell proliferation analysis by 4 h EdU labeling at P35 after 2-weeks of Oxytocin treatment, 15–20 slices from 3 to 4 mice per group, scale bar: 200 μm. One-way analysis of variance (ANOVA) were performed for statistical analysis (*Aurs2^fl/fl + Saline^* vs. *Aurs2^fl/fl;Emx1-Cre^* + Saline, *p* < 0.001; *Aurs2^fl/fl + Saline^* vs. *Aurs2^fl/fl;Emx1^-Cre* + Oxytocin, *p* < 0.001; *Aurs2^fl/fl;Emx1-Cre^* + Saline vs. *Aurs2^fl/fl;Emx1-Cre^* + Oxytocin, *p* = 0.014). **(C–D)** Representative heat maps and statistical analysis of three-chamber social interaction test in different genotypes and treatment groups, 9–10 male mice per group. Data were shown as means ± SEM. Two-tailed Student’s *t*-test and two-way analysis of variance (ANOVA) were performed for statistical analysis (Trial 1: Main effect of genotypes *p* = 0.931; Main effect of S1/EM *p* < 0.001; Main effect of S1/EM × genotype *p* = 0.635; Trial 2: Main effect of genotypes *p* = 0.471; Main effect of S1/S2 *p* = 0.017; Main effect of S1/S2 × genotype *p* = 0.008). *p < 0.05, **p < 0.01 and ***p < 0.001; n.s., no significance.

First, oxytocin administration was conducted to stimulate the expansion of DG NSCs in *Auts2^fl/fl;Emx1-Cre^* mice. At the age of 3 weeks, mice were given intraperitoneal injection of oxytocin daily for 2 weeks, and then 5-Ethynyl-2′-deoxyuridine (EdU) labeling was used to detect the cell proliferation of DG after oxytocin treatment (Figure 1A). The result showed that the EdU^+^ cells in SGZ of saline group in *Auts2^fl/fl;Emx1-Cre^* mice was decreased compared with *Auts2^fl/fl^* mice. However, the oxytocin administration significantly increased EdU^+^ cells in *Auts2^fl/fl;Emx1-Cre^* mice (Figure 1B). The three-chamber social interaction test was performed at 6 weeks of age to evaluate the improvement of social ability and social novelty preference in *Auts2^fl/fl;Emx1-Cre^* mice. In the social ability test (Trial 1), three groups of mice showed preference toward Stranger1, with no significant difference among them. While the *Auts2^fl/fl;Emx1-Cre^* mice with oxytocin administration spent more time in social interaction with Stranger2 in Trial 2 when compared with the *Auts2^fl/fl;Emx1-Cre^* mice in saline group (Figures 1C,D). These results suggested that the oxytocin repeated treatment increases the number of newborn neurons in DG and improves the impaired social recognition in *Auts2^fl/fl;Emx1-Cre^* mice. Furthermore, we calculated the length of suprapyramidal blade/infrapyramidal blade as well as DG area to determine whether oxytocin administration could reverse the DG hypoplasia of *Auts2^fl/fl;Emx1-Cre^* mice. However, the results showed that there was no statistical differences between saline and oxytocin administration groups (Supplementary Figure 1A).

### Post-weaning enriched environment feeding promotes the proliferation of NSCs in DG and improves the social recognition deficit in *Auts2^fl/fl;Emx1-Cre^* mice

**Figure 2:**
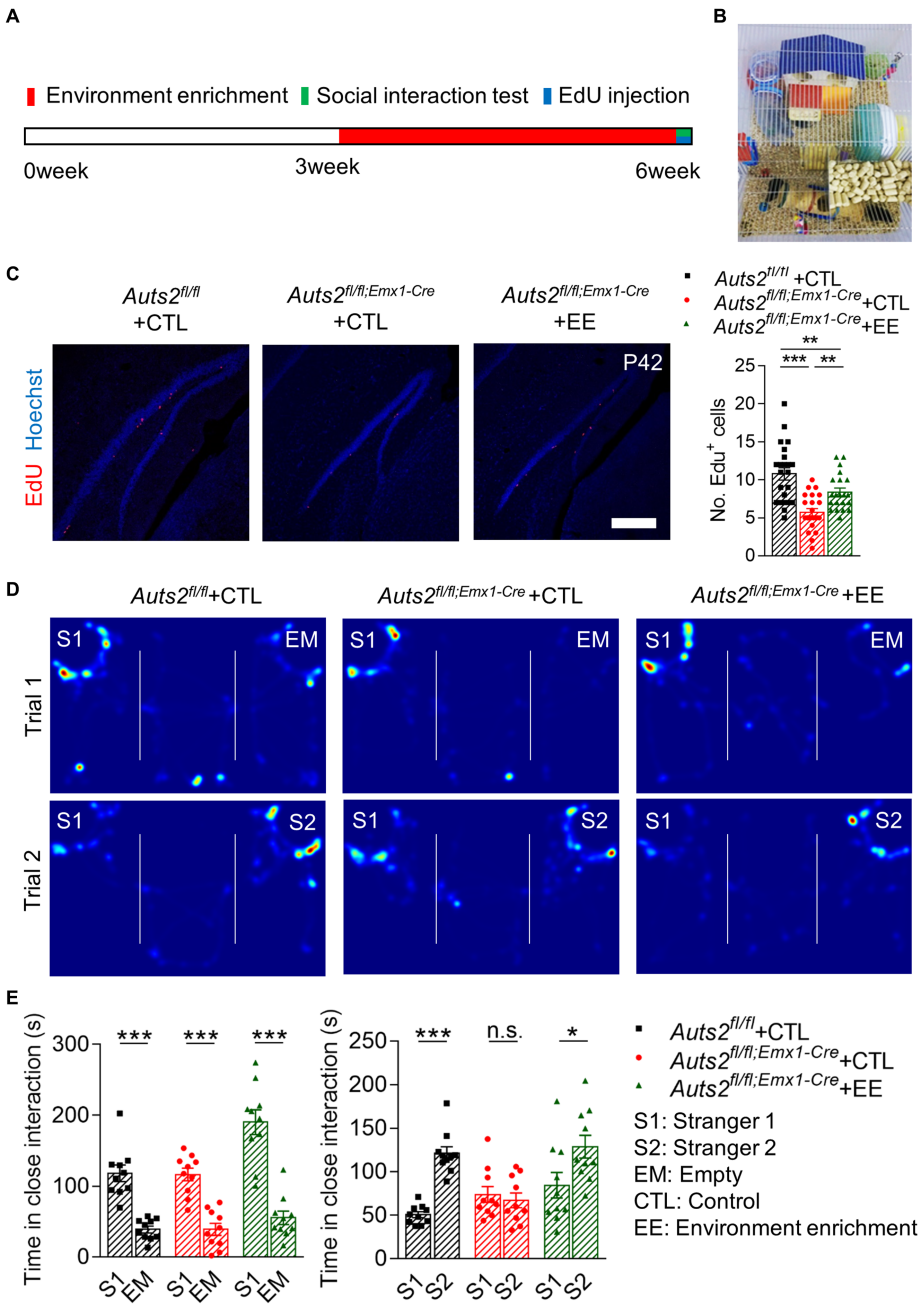
Post-weaning enriched environment feeding promotes the proliferation of NSCs in DG and improves the social recognition deficit in *Auts2^fl/fl;Emx1-Cre^* mice **(A)** Experiment strategy of environment enrichment, proliferation analysis and social behavioral test. **(B)** Picture of enriched environment feeding condition. **(C)** Cell proliferation analysis by 4 h EdU labeling at P42 after 3 weeks of environment enrichment rearing, 15–20 slices from 3 to 4 mice per group, scale bar: 200 μm. One-way analysis of variance (ANOVA) were performed for statistical analysis (*Aurs2^fl/fl^* + CTL vs. *Aurs2^fl/fl;Emx1-Cre^* + CTL, *p* < 0.001; *Aurs2^fl/fl^* + CTL vs. *Aurs2^fl/fl;Emx1-Cre^* + EE, *p* < 0.001; Aurs2fl/fl;Emx1-Cre + CTL vs. Aurs2fl/fl;Emx1-Cre + EE, *p* = 0.734). **(D–E)** Representative heat maps and Statistical analysis of three-chamber social interaction test in different genotypes and treatment groups, 10–11 male mice per group. Data were shown as means ± SEM. Two-tailed Student’s *t*-test and two-way analysis of variance (ANOVA) were performed for statistical analysis (Trial 1: Main effect of genotype, *p* < 0.001; Main effect of S1/EM, *p* < 0.001; Main effect of S1/EM × genotype, *p* = 0.014; Trial 2: Main effect of genotype, *p* = 0.003; Main effect of S1/S2, *p* < 0.001; Main effect of S1/S2 × genotype, *p* = 0.001). *p < 0.05, **p < 0.01 and ***p < 0.001; n.s., no significance.

The *Auts2^fl/fl;Emx1-Cre^* mice were raised in cages with enriched environment post-weaning to induce the proliferation of DG NSCs. After 3 weeks of environmental enrichment stimulation, the behavioral tests and EdU injection were performed at the age of 6 week (Figures 2A,B). The number of EdU^+^ cells in DG SGZ of *Auts2^fl/fl;Emx1-Cre^* mice was decreased compared with *Auts2^fl/f^* mice, while the number of which in the environment enrichment group of *Auts2^fl/fl;Emx1-Cre^* mice is much more than control group (Figure 2C). In the social behavior, the *Auts2^fl/fl;Emx1-Cre^* mice of control group showed normal preference toward S1 in Trial 1 and no preference between S1 and S2 in Trial 2. The *Auts2^fl/fl;Emx1-Cre^* mice in environment enrichment group showed significant social preference toward S2 in Trial 2 of three-chamber social interaction assay when compared with the control group. In addition, the *Auts2^fl/fl;Emx1-Cre^* mice in environment enrichment group showed better social ability in the first stage of the behavior test, showing more interesting in stranger 1 compare with control group of both *Auts2^fl/fl^* and *Auts2^fl/fl;Emx1-Cre^* mice in Trial 1 (Figures 2D,E). These results suggested that environment enrichment also increases the number of newborn neurons in DG and improves both of the social ability and social novelty recognition ability of *Auts2^fl/fl;Emx1-Cre^* mice. We also calculated the length of suprapyramidal blade, length of infrapyramidal blade and area of DG but no significant difference between control and environment enrichment groups was found (Supplementary Figure 1B).

### Cdk4 and CyclinD1 expression in DG NSCs expands newborn neurons and restores impaired social novelty recognition in *Auts2^fl/fl;Emx1-Cre^* mice

**Figure 3:**
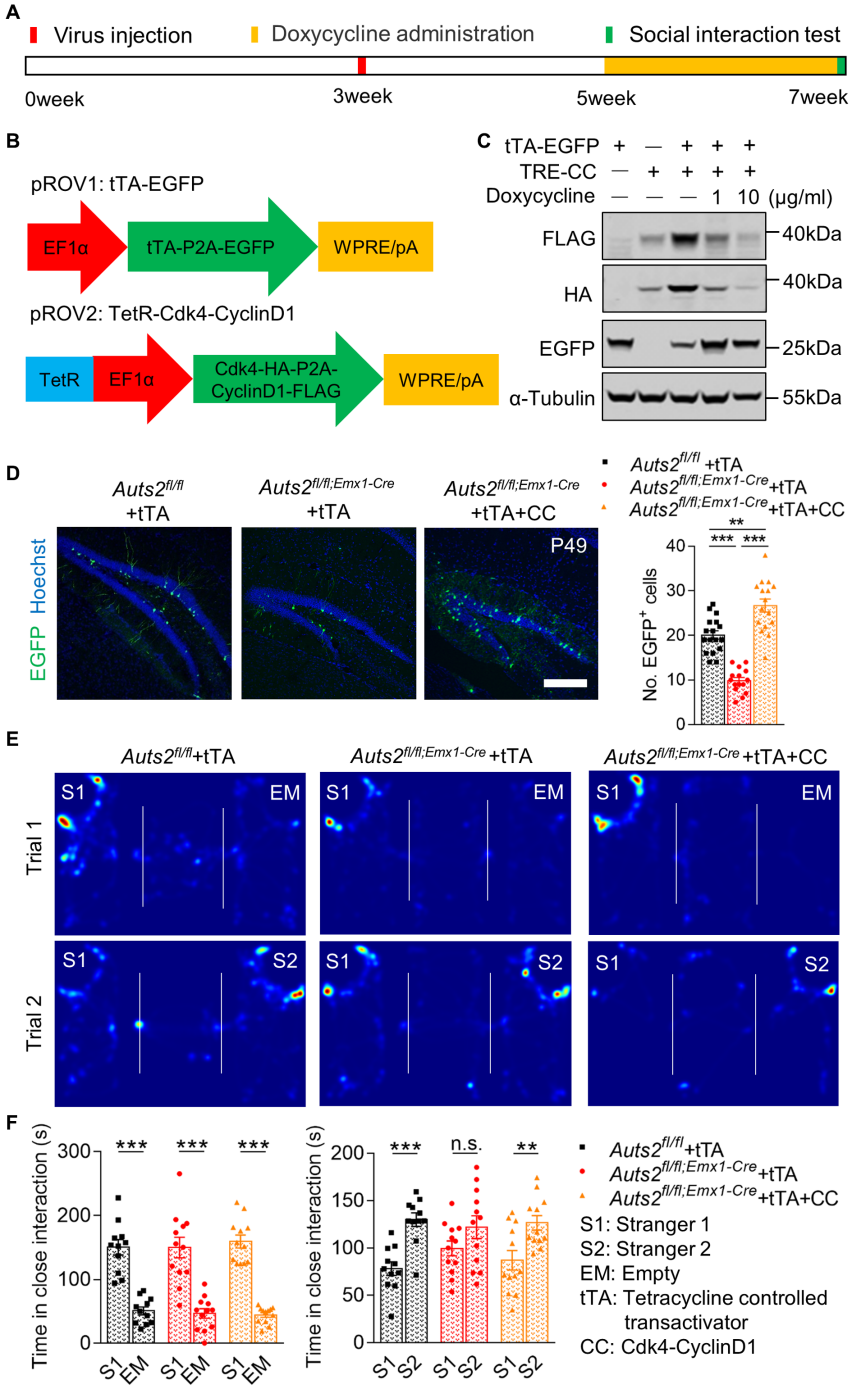
Cdk4 and CyclinD1 expression in DG NSCs expands newborn neurons and restores impaired social novelty recognition in *Auts2^fl/fl;Emx1-Cre^* mice. **(A)** Experiment strategy of Virus injection, doxycycline administration and social interaction test. **(B)** Schematic of the Tet-Off system and Cdk4-cyclinD1 expression pattern in retrovirus. **(C)** Protein levels of FLAG-labeled CyclinD1, HA-labeled Cdk4 and EGFP in HEK cell of different groups; α-Tubulin was used as internal reference. **(D)** Representative images and statistical analysis of retrovirus expression in different genotypes and treatment groups after two-week of proliferation and two-week of differentiation. Fifteen to twenty slices from 3 to 4 mice per group, scale bar: 200 μm. One-way analysis of variance (ANOVA) were performed for statistical analysis (*Aurs2^fl/fl^* + tTA vs. *Aurs2^fl/fl;Emx1-Cre^* + tTA, *p* < 0.001; *Aurs2^fl/fl^* + tTA vs. *Aurs2^fl/fl;Emx1-Cre^* + tTA + CC, *p* = 0.009; *Aurs2^fl/fl;Emx1-Cre^* + Saline vs. *Aurs2^fl/fl;Emx1-Cre^* + Oxytocin, *p* < 0.001). **(E–F)** Representative heat maps and statistical analysis of social interaction test in different genotypes and treatment groups, 11–12 male mice per group. Data were shown as means ± SEM. Two-tailed Student’s *t*-test and two-way analysis of variance (ANOVA) were performed for statistical analysis (Trial 1: Main effect of genotype, *p* = 0.358; Main effect of S1/EM, *p* < 0.001; Main effect of S1/EM × genotype, *p* = 0.595; Trial 2: Main effect of genotype, *p* = 0.858; Main effect of S1/S2, *p* < 0.001; Main effect of S1/S2 × genotype, *p* < 0.001). *p < 0.05, **p < 0.01 and ***p < 0.001; n.s., no significance.

Retrovirus was selected to specifically express Cdk4 and CyclinD1 in hippocampal NSCs and induce their proliferation in *Auts2^fl/fl;Emx1-Cre^* mice. In order to achieve time-specific expression of Cdk4 and CyclinD1, we introduced the Tet-off system. As shown in Figure 3A, we constructed two retrovirus, with tetracycline controlled transactivator (tTA) and EGFP expression in one, and tetracycline response (tetR) element-driven HA-tagged Cdk4 and FLAG-tagged CyclinD1 in another (Figure 3B). The expression and silencing of Cdk4 and CyclinD1 were verified by using western blotting in HEK cell line with or without Doxycycline administration (Figure 3C). These two retroviruses were injected together into bilateral DG in *Auts2^fl/fl;Emx1-Cre^* mice at P21. For the control, retrovirus expressing tTA-EGFP was injected into *Auts2^fl/fl^* and *Auts2^fl/fl;Emx1-Cre^* mice alone. After 2 weeks, the Doxycycline was administered through drinking water in all three groups of mice to stop the Cdk4 and CyclinD1 expression, so that the newborn cells could undergo subsequent differentiation. The number of EGFP^+^ newborn neurons in *Auts2^fl/fl;Emx1-Cre^* mice of tTA-expression group was decreased compared with that of *Auts2^fl/fl^* mice, while the number of which in the Cdk4-CyclinD1 overexpression group was significantly increased another 2 weeks later (Figure 3D). Three-chamber social interaction assay also showed that the social recognition deficit in *Auts2^fl/fl;Emx1-Cre^* mice were rescued in Cdk4-CyclinD1 overexpression group (Figures 3E,F). These results indicated that the expansion of DG newborn neurons in by Cdk4-CyclinD1 overexpression improves the impaired social novelty recognition in *Auts2^fl/fl;Emx1-Cre^* mice. However, the DG hypoplasia cannot be reversed (Supplementary Figure 1C).

## Discussion

**Figure 4:**
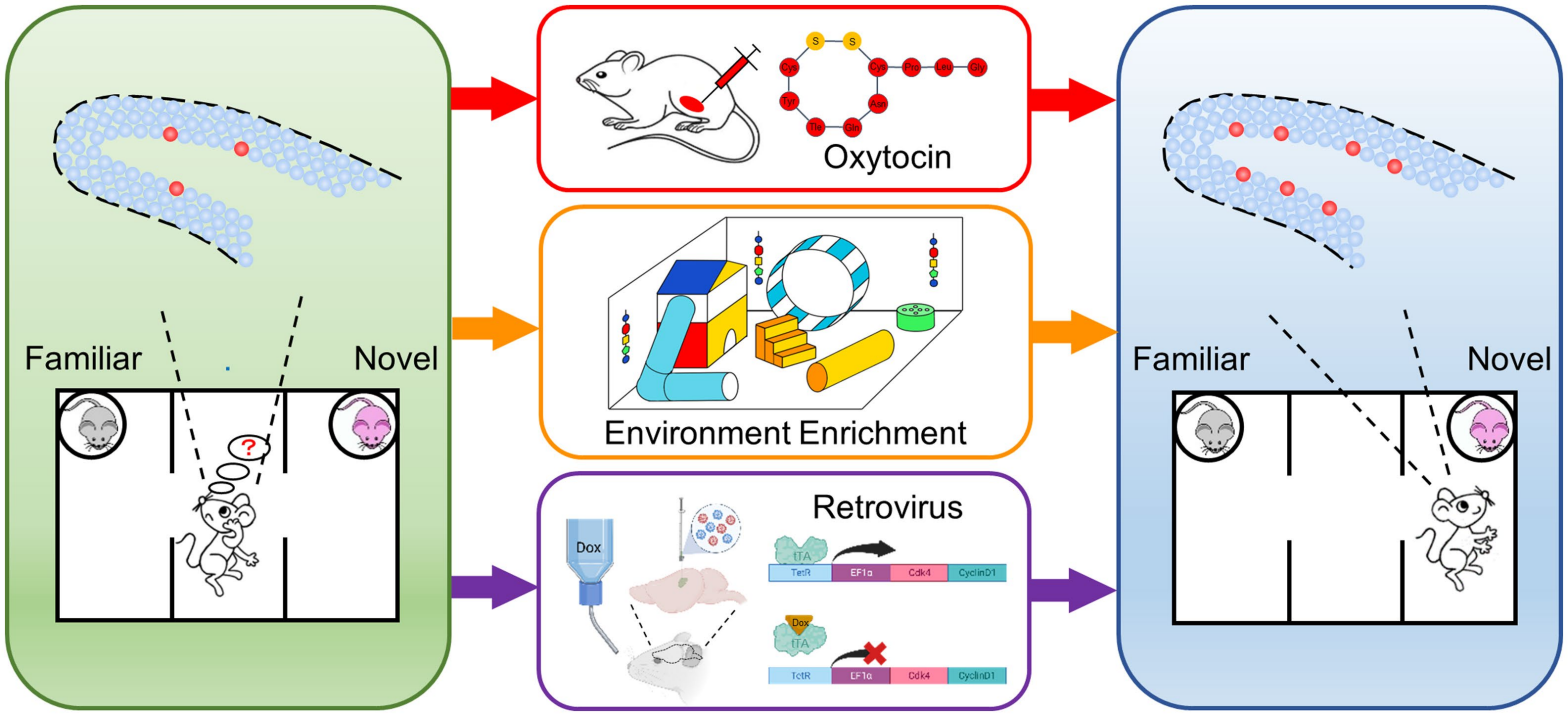
Schematic drawings of representative experimental procedures for increasing neurogenesis in DG and restoring impaired social novelty recognition *Auts2^fl/fl;Emx1-Cre^* mice showed DG hypoplasia, decreased of neural stem cell and social novelty recognition deficit **(left)**. Three approaches including repeated oxytocin administration, feeding in environment enrichment and genetic overexpression of cyclin-dependent kinase 4 (Cdk4) and CyclinD1 were performed **(middle)**. The expansion of neural stem cells and the improvement of social recognition deficit were identified after the treatments **(right)**.

ASDs are a heterogeneous group of neurodevelopmental conditions, which are difficult to prevent and diagnose at embryonic or early postnatal stage. Most of the patients showed abnormal neurodevelopmental phenotypes when clinically diagnosed, which is hard to directly reverse. Therefore, it is necessary to explore new approaches for treatment. In previous studies, we found that *Auts2^fl/fl;Emx1-Cre^* mice showed DG hypoplasia and social recognition deficit. In this study, we found that repeated oxytocin administration, feeding in environment enrichment and genetic modification-based neural stem cell expansion improved the impaired social novelty recognition in *Auts2^fl/fl;Emx1-Cre^* mice (Figure 4). Our results provided evidence that the expansion of adult-newborn neurons can improve the ASDs-related social recognition deficit.

The hippocampus-dependent learning task could be impaired if adult-generated neurons decreased in DG (27–29). When the number of newborn neurons in DG is increased by environment enrichment (30) or exercise (31, 32), the learning tasks are improved, suggesting that SGZ newborn neurons can integrate into DG granular cells and participate in memory, learning, and cognitive flexibility (33–36). Although DG hypoplasia cannot be reversed by the above three approaches in the current study, the significantly increased number of newborn granular cells (GCs) could effectively improve the impaired social novelty preference of *Auts2^fl/fl;Emx1-Cre^* mice. It might be related to the integration of newborn neurons into the neural circuit of GCs in DG (29), which forming glutamatergic terminals on hilar or CA3 pyramidal cells (37), and improving the defect of DG to CA3 transmission in *Auts2^fl/fl;Emx1-Cre^* mice.

Although the three strategies can promote DG newborn neurons and rescue social recognition defects, the mechanisms might not be completely the same. Oxytocin plays a role as neuromodulator through its receptor which distributed in the brain (38). It is considered as a possible medication for the treatment of autism both in human (39–41) and rodent (42–44). Rat study showed that repeated administration of oxytocin can increase the number of newborn neurons in DG (22). Lin et al. showed that oxytocin administration increased the number of newborn neurons in DG through the oxytocin receptor in CA2/CA3 region (45). Pan et al. reported that the synaptic plasticity of the neural circuit in the hippocampus could also be improved by repeated administration of oxytocin (46). In addition, its possible that oxytocin modulates these processes indirectly by acting on oxytocin–responsive cells within brain regions that provide afferent input to the dentate gyrus such as the amygdala (47) or cortices (48), indicating that the oxytocin may act in multiple ways to induce the neurogenesis of DG.

Studies on environment enrichment showed that its influence on the number of newborn neurons is generated by multiple factors (49). Among them, the physical components (less partners with toys) of environment enrichment affect the proliferation of hippocampal neurons, and the social components (more partners without toys) affect the survival of hippocampal proliferating neurons (50). If the mitosis was inhibited by medication AraC, the effect of environment enrichment will be weakened (23). Interestingly, environment enrichment could increase the expression oxytocin in the hypothalamic paraventricular (PVN) and supraoptic (SON) nuclei (51). If oxytocin receptor inhibitor was given during environment enrichment, the effect of which could also be weakened (52). It means that the environment enrichment might promote the release of oxytocin and through which stimulate the cell proliferation. In addition, here we also found that the environment enrichment not only improved the impaired social novel recognition of *Auts2^fl/fl;Emx1-Cre^* mice, but also increased their social ability. The reason might be that environment enrichment could not only facilitate the generation of newborn neurons in DG, but also increase the neurogenesis in the Olfactory Bulb (OB) granule cell layer (GCL), which is fundamental in allowing mice to recognize conspecifics (23).

Although the oxytocin and environmental stimuli could improve the social behavior in mice (23, 44), the influence of these approaches covered the whole body and nervous system. It is not clear whether stimulating the proliferation of DG stem cells alone can rescue the social deficit. Thus we triggered the neurogenesis in DG by genetic modulation (24). Previous literature reports that overexpression of the Cdk4 (cyclin-dependent kinase 4)–CyclinD1 complex in the adult mouse hippocampus cell-autonomously increases the expansion of neural stem and progenitor cells while inhibiting neurogenesis. Here we combined Cdk4-CyclinD1 system and Tet-off system and apply it to the treatment of social behavior abnormalities in an autistic mouse model for the first time. Compared with the oxytocin and environment enrichment, the expression of Cdk4 and CyclinD1 in NSCs of DG SGZ region by genetic method to induce the proliferation of NSCs is a more direct method to increase the number of newborn neurons. It suggested that increasing the number of newborn neurons can indeed reverse the deficit of social recognition behavior. However, owing to ethical concerns and safety, this method may be not feasible for patients at present. While oxytocin administration and enriched environment stimulation showed more advantages in transformation. For example, although the efficacy is controversial, some clinical evidence showed that functional training (53, 54) and nasal oxytocin (39) can effectively improve the social cognition of autistic patients, the mechanism of which might be the enhancement of neurogenesis in hippocampus. However, whether the therapeutic response is related to the abnormal development or dysfunction of DG needs further investigation in the future.

ASDs are a group of neurodevelopmental disorders with highly heterogeneous. DG hypoplasia might be a critical pathological phenotype observed in some individuals of ASDs (9, 55), which involves in the social deficits by affecting hippocampal function (56–59). Our research provided evidence that the expansion of DG newborn neurons reversed the abnormal social recognition behavior in a mouse model with DG hypoplasia. It is highly possible that the three manipulations could apply to autistic individuals with DG hypoplasia. However, the effectiveness of these approaches on other subclasses of ASDs requires further experiments to explore and verify.

In summary, we successfully improved the social novelty recognition deficit in a mouse model of autism with DG hypoplasia by three methods based on the increase of neurogenesis and newborn neurons. In addition, the combination of Cdk4-CyclinD1 system and Tet-off system were first employed to spatiotemporally control the proliferation and differentiation of NSCs by retrovirus delivery in DG. Our findings might provide a novel strategy to treat social deficits in patients with abnormal development or dysfunction of hippocampus.

## Data availability statement

The original contributions presented in the study are included in the article/Supplementary material, further inquiries can be directed to the corresponding authors.

## Ethics statement

The animal study was reviewed and approved by the Animal Ethical Committee of Peking University Health Center.

## Author contributions

JL, DZ, LW, and WY contributed to the conception of the study. HM and QL performed the experiment. HM, QL, and JL contributed significantly to analysis and manuscript preparation. HM and JW performed the data analyses and wrote the manuscript. XS, JL, DZ, LW, and WY helped perform the analysis with constructive discussions. All authors contributed to the article and approved the submitted version.

## Funding

This work was supported by grants from Key Realm R&D Program of Guangdong Province (No. 2019B030335001) and the National Natural Science Foundation of China (Nos. 81825009, 82071541, 81971283, 82271576, and 82101570).

## Conflict of interest

The authors declare that the research was conducted in the absence of any commercial or financial relationships that could be construed as a potential conflict of interest.

## Publisher’s note

All claims expressed in this article are solely those of the authors and do not necessarily represent those of their affiliated organizations, or those of the publisher, the editors and the reviewers. Any product that may be evaluated in this article, or claim that may be made by its manufacturer, is not guaranteed or endorsed by the publisher.
