# Supplementary figures and images for: The expansion of newborn neurons in hippocampus improves social recognition deficit in a mouse model of autism
Source: Front Psychiatry. 2023 May 5;14:1162179. doi: 10.3389/fpsyt.2023.1162179 (PMC10196005; doi:10.3389/fpsyt.2023.1162179)

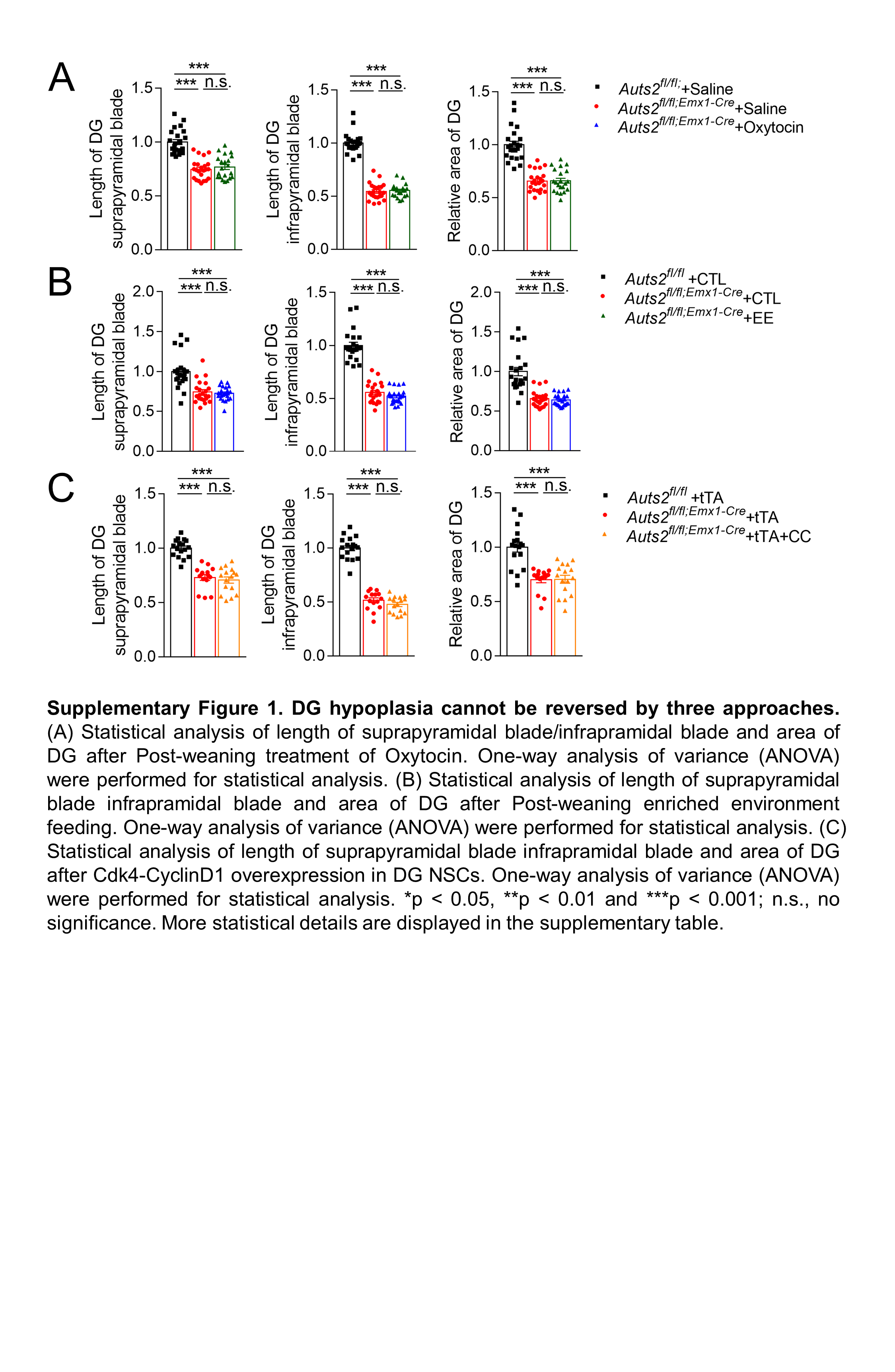

Supplement: Supplementary file 2 [file Image_1.TIF]

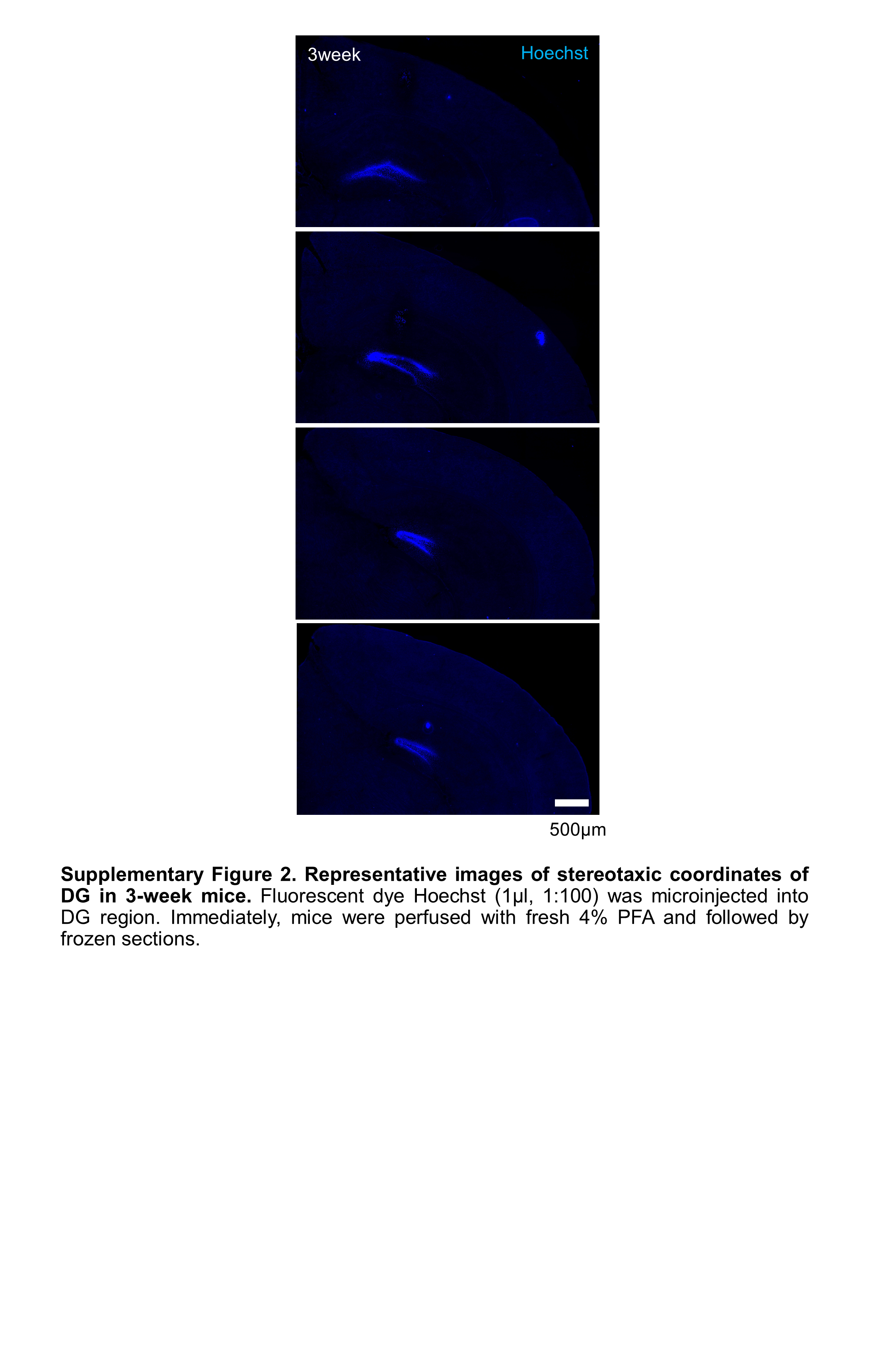

Supplement: Supplementary file 3 [file Image_2.TIF]
